# Note on the Thermal Degradation of Polytetrafluoroethylene as a First-Order Reaction

**DOI:** 10.6028/jres.064A.050

**Published:** 1960-12-01

**Authors:** S. L. Madorsky, S. Straus

## Abstract

Additional experiments on the rates of thermal degradation of polytetrafluoroethylene in a vacuum confirm an earlier conclusion that a first-order rate law is involved in the degradation reaction.

In a study made by Madorsky, Hart, Straus, and Sedlak^1^ on the rates and activation energy of thermal degradation of polytetrafluoroethylene in a vacuum, two methods were employed: a gravimetric method, using a very sensitive tungsten spring balance in a vacuum system to measure the rate of loss of weight of the degrading sample, and a pressure method, using a multiplying manometer to measure the pressure of the C_2_F_4_ formed in the reaction. The material that was used was in the form of a tape 0.07 mm thick. Weight of the sample was about 7 mg in the gravimetric experiments and 5 to 306 mg in the pressure experiments.

The rates obtained by the gravimetric method are reproduced in figure 1, plotted as a function of percentage loss of weight of the sample for 480, 490, 500, and 510 °C. The initial rates were obtained by extrapolating the rate curves to the ordinate. The rate curves beyond the initial 5 to 18 percent loss of weight of the sample are straight lines, and when extended to the right they approach near the zero rate at 100 percent volatilization. The rates obtained by the pressure method were studied at 10 different temperatures ranging from 423.5 to 513.0 °C. Logarithms of the initial rates obtained by both methods are shown in figure 2 plotted against the inverse of absolute temperature.^2^ From the Arrhenius equation the slope of the resulting straight line indicates an activation energy of 80.5 kcal/mole. From the appearance of the curves in figure 1 it seemed logical to conclude that the reaction involved in the thermal degradation of polytetrafluoroethylene in a vacuum follows a first-order law.

At a later date Wall and Michaelson^3^ studied the rate of thermal degradation of polytetrafluoroethylene at 460 °C in a stream of nitrogen. They used a gravimetric method by heating 1-g samples of a powdered material and weighing the residues at intervals. They state that below about 480 °C the reaction is zero order, whereas above 510 °C they concede it is first order.

In view of this result by Wall and Michaelson, it was deemed advisable to check further on the rate order involved in the thermal degradation of this material in a vacuum. Although experiments by the pressure method were carried out in our earlier work at temperatures below 480 °C, the extent of volatilization was at most only 6.4 percent. Degradation had not been carried far enough to determine accurately whether the percentage loss versus time plots were straight or curved lines, i.e., whether the indicated reaction is of zero or first order. Rate experiments were therefore carried out by the weight method at lower temperatures, namely at 460, 475, and 485 °C, and the results are shown in figure 3, where percentage loss of weight is plotted against time. The curves are definitely not straight lines, as would have been the case if the reaction had followed a zero order.

In our previous work (see footnote 1) the rates were obtained by plotting the slopes between two neighboring experimental points in the volatilization-time plots. In the present work the slopes were calculated from the curves shown in figure 3, and the resulting rate curves based on these calculations are shown in figure 4. The same type of rate curves were obtained as in the earlier work. Values obtained for the initial rates at these three temperatures fit nicely into the Arrhenius plot, as shown by the squares in figure 2.

The present work therefore confirms our earlier conclusion that the degradation of polytetrafluoroethylene in a vacuum follows a first-order rate law, where the rate of volatilization, based on the sample, is directly proportional to the amount of residue.

## Figures and Tables

**Figure 1:**
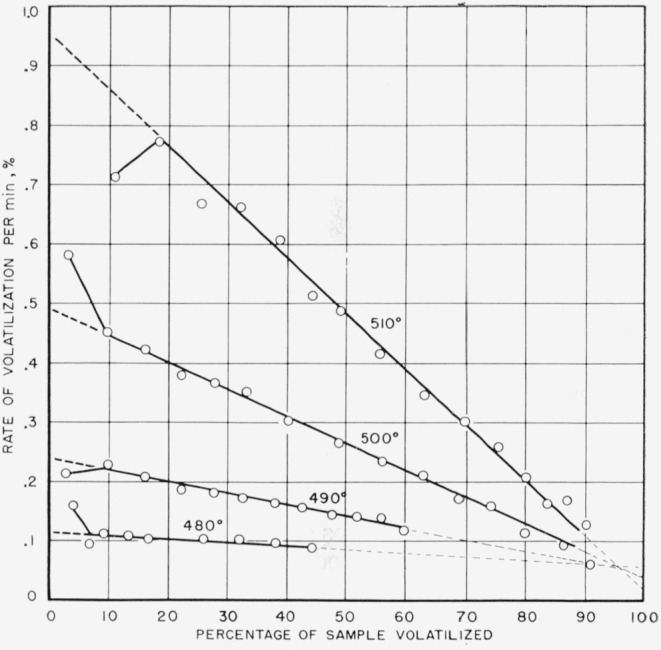
Rate of thermal degradation of polytetrafluoroethylene by the weight method as a function of percentage volatilization.

**Figure 2:**
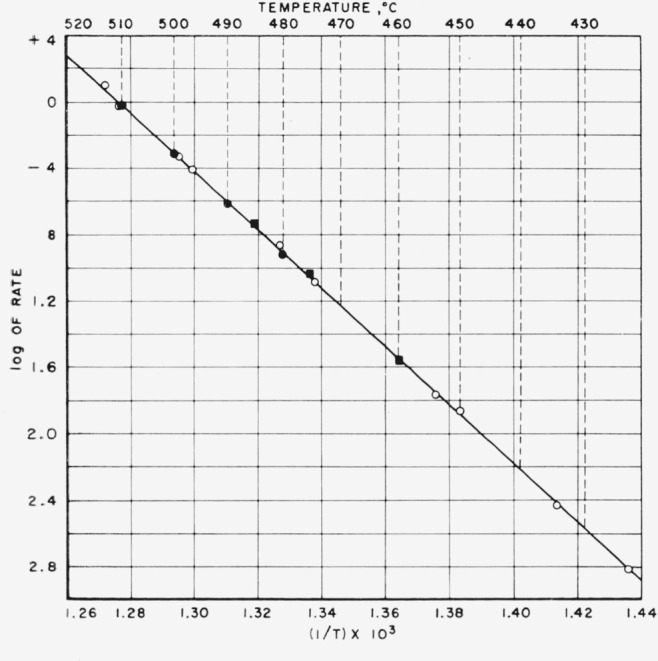
Activation energy slope for thermal degradation of polytetrafluoroethylene. ●—weight method (see footnote 1) ○—pressure method (see footnote 1) ■—present work.

**Figure 3:**
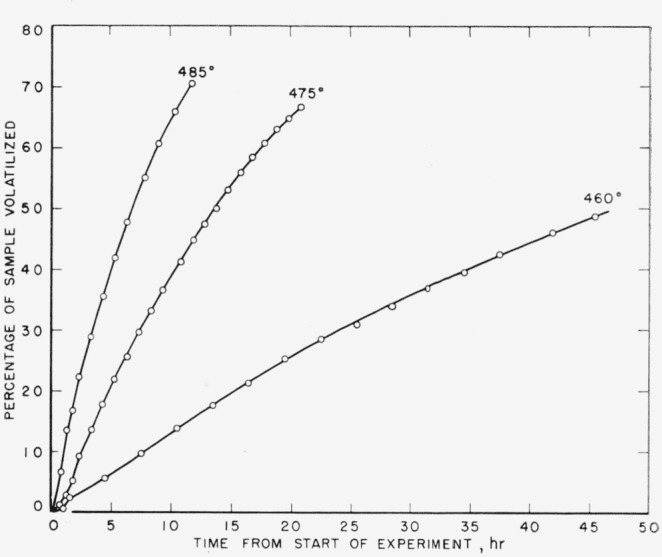
Pyrolysis of polytetrafluoroethylene at low temperatures.

**Figure 4:**
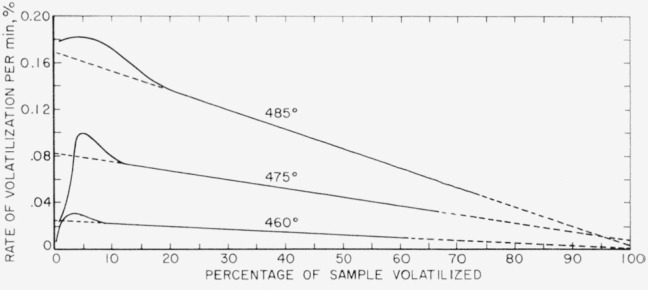
Rates of thermal degradation of polytetraduoroethylene at low temperatures.

## Footnotes

1 S. L. Madorsky, V. E. Hart, S. Straus, and V. A. Sedlak, *J. Research NBS* 51, 327 (1953) RP2461.

2 In the earlier paper (see footnote 1), the abscissa of a similar graph (fig. 5) was incorrectly labelled log (1/*T*)×10^3^, whereas it should have been (1/*T*)×10^3^.

3 L. A. Wall and J. D. Michaelson, *J. Research, NBS* 56, 27 (1956) RP2644.

